# Leukodepleted versus nonleukodepleted red blood cell transfusion in septic patients: a microcirculatory vision

**DOI:** 10.1186/cc13811

**Published:** 2014-04-01

**Authors:** Sebastien Tanaka, Anatole Harrois, Jacques Duranteau

**Affiliations:** 1 AP-HP, Service d’Anesthésie-Réanimation, Hôpitaux Universitaires Paris-Sud, Université Paris-Sud, Hôpital de Bicêtre, Le Kremlin-Bicêtre 94275, France

## Abstract

Storage time and residual leukocytes in red blood cell (RBC) units may be deleterious by increasing the accumulation of leukocyte-derived cytokines and by raising the adhesion of RBCs to endothelium. Leukodepleted RBC transfusion may reduce the incidence of infection and organ dysfunction. However, the influence of leukodepletion on microcirculation remains not well defined in ICU patients. In this context, an original study in a previous issue of *Critical Care* emphasizes the microcirculatory effects of transfusion of leukodepleted RBCs (post-storage leukoreduction) or nonleukodepleted RBCs in septic patients. This study suggests a positive rheological impact of leukodepleted RBCs in septic patients with an increase in sublingual microvascular flow and perfused vessel density. Given the variability in the microvascular response to RBC transfusion in individual patients, there is a need for monitoring the microcirculation to guide transfusion in patients with sepsis rather than deciding to transfuse RBCs according to an arbitrary hemoglobin level. Further studies to identify the microvascular response to RBC transfusion in ICU patients are warranted.

## 

Approximately one-third of critically ill patients receive a blood transfusion at one time or another during their stay in the ICU. The expected response after blood transfusion in ICU patients is an increase in macrovascular oxygen delivery associated with an improvement of tissue oxygenation. Although microcirculation is the cornerstone of gas and nutritive exchanges for tissues, only few studies have reported the microcirculatory effects of transfusion – especially in septic patients. In addition to sepsis-induced alteration of microcirculation, the rheologic properties of the transfused red blood cells (RBCs) may be altered during RBC storage.

As a restrictive versus a liberal strategy transfusion in ICU, the age of RBCs or the use of leukodepleted RBCs remain controversial and still debated. Leukodepletion policies strongly differ from one country to another. Prolonged storage time and residual leukocytes in RBC units may be deleterious by increasing the accumulation of leukocyte-derived cytokines and by raising the adhesion of RBCs to endothelium. Leukodepleted RBC transfusion may reduce the incidence of infection and organ dysfunction. However, the influence of leukodepletion on microcirculation remains not well defined in ICU patients.

In this context, an original study in a previous issue of *Critical Care* emphasizes the microcirculatory effects of leukodepleted RBC transfusion or nonleukodepleted RBC transfusion in septic patients [1]. Microcirculatory perfusion was significantly improved in the leukodepleted blood group compared with the nonleukodepleted blood group concerning the microcirculatory flow index and the blood flow velocity. The within-group analysis showed that the sublingual microcirculatory flow index, perfused vessel density, proportion of perfused vessels (PPV) and De Backer score increased in septic patients who received leukodepleted RBCs, but not in those who received nonleukodepleted RBCs. In addition, a decrease in blood flow velocity was observed after nonleukodepleted RBC transfusion. Interestingly, syndecan-1, which happens to be a promising marker of endothelial dysfunction [2], increased in the nonleukodepleted group after transfusion, suggesting a fragmentation of the endothelial glycocalyx in this group.

*In vitro* studies have previously reported a direct effect of white blood cells or their byproducts on RBC deformability and adhesiveness to microvascular endothelium [3,4], but the impact of leucocytes in storage-induced changes in RBC adherence was not evaluated in septic patients. Despite limitations (low number of patients, heterogeneous population of septic patients with unbalanced severity between the two groups), the study by Donati and colleagues suggests a positive rheological impact of leukodepleted RBCs in septic patients [1]. In a previous study, in a larger population of severe sepsis patients, Sakr and colleagues observed that leukodepleted RBC transfusion had no straightforward effect on sublingual microcirculation [5]. These authors found no changes in PPV and perfused vessel density after transfusion of 1 to 2 units of leukodepleted RBCs. The same study highlighted the fact that there is a considerable interindividual variability in the microvascular response after transfusion. Improvement in sublingual microvascular perfusion was observed in patients with an altered sublingual microvascular perfusion at baseline. In contrast, when the sublingual microvascular perfusion was preserved at baseline, RBC transfusion altered the PPV. The study by Donati and colleagues confirmed that changes in PPV after blood transfusion were negatively correlated to baseline PPV [1].

The main point to note is that RBCs can only improve altered microcirculation. When microvascular perfusion is already restored or preserved before transfusion, the need for transfusion is reduced. In addition, the microvascular response to RBC transfusion cannot be predicted from macrovascular hemodynamic parameters and systemic hemoglobin [1,5]. This point illustrates the need for microcirculation monitoring to guide transfusion in patients with sepsis rather than deciding to transfuse RBCs according to an arbitrary hemoglobin level.

Given the variability in the microvascular response to RBC transfusion in individual patients, further research in this area might try to include more patients in a well-defined population. It appears also important to evaluate, in parallel to microcirculation perfusion, the effect of transfusion on cardiac output and oxygen delivery to properly compare changes in systemic oxygen delivery with changes in microvascular parameters. In addition, the time from sepsis onset to transfusion should be taken into consideration. Ospina-Tascon and colleagues have reported a difference between microvascular responses early and late in the course of sepsis despite similar increases in global hemodynamic parameters [6].

Understanding the interactions of transfused RBCs with microcirculation is crucial to optimize tissue oxygenation with RBC transfusion and to improve RBC storage. Further studies to identify the microvascular response to RBC transfusion in ICU patients are warranted.

### Abbreviations

PPV: Proportion of perfused vessels; RBC: Red blood cell.

### Competing interests

The authors declare that they have no competing interests.

### Authors' contributions

All authors contributed equally to this work. All authors read and approved the final manuscript.
